# The Academic Medical Center Linear Disability Score (ALDS) item bank: item response theory analysis in a mixed patient population

**DOI:** 10.1186/1477-7525-3-83

**Published:** 2005-12-29

**Authors:** Rebecca Holman, Nadine Weisscher, Cees AW Glas, Marcel GW Dijkgraaf, Marinus Vermeulen, Rob J de Haan, Robert Lindeboom

**Affiliations:** 1 Department of Neurology, Academic Medical Center, Amsterdam, The Netherlands; 2 Department of Educational Measurement, University of Twente, Enschede, The Netherlands; 3 Department of Clinical Epidemiology and Biostatistics, Academic Medical Center, Amsterdam, The Netherlands; 4 Department of Anesthesiology, Academic Medical Center, Amsterdam, The Netherlands

## Abstract

**Background:**

Currently, there is a lot of interest in the flexible framework offered by item banks for measuring patient relevant outcomes. However, there are few item banks, which have been developed to quantify functional status, as expressed by the ability to perform activities of daily life. This paper examines the measurement properties of the Academic Medical Center linear disability score item bank in a mixed population.

**Methods:**

This paper uses item response theory to analyse data on 115 of 170 items from a total of 1002 respondents. These were: 551 (55%) residents of supported housing, residential care or nursing homes; 235 (23%) patients with chronic pain; 127 (13%) inpatients on a neurology ward following a stroke; and 89 (9%) patients suffering from Parkinson's disease.

**Results:**

Of the 170 items, 115 were judged to be clinically relevant. Of these 115 items, 77 were retained in the item bank following the item response theory analysis. Of the 38 items that were excluded from the item bank, 24 had either been presented to fewer than 200 respondents or had fewer than 10% or more than 90% of responses in the category 'can carry out'. A further 11 items had different measurement properties for younger and older or for male and female respondents. Finally, 3 items were excluded because the item response theory model did not fit the data.

**Conclusion:**

The Academic Medical Center linear disability score item bank has promising measurement characteristics for the mixed patient population described in this paper. Further studies will be needed to examine the measurement properties of the item bank in other populations.

## Background

Functional status is now seen as an important determinant of patients' quality of life and a wide variety of instruments have been developed [1]. Instruments for quantifying functional status tend to have a fixed length and administer all items to the whole group of patients under scrutiny. Currently, interest is moving towards the more flexible framework offered by item banks in conjunction with item response theory. An item bank is a collection of items, for which the measurement properties of each item are known [2,3]. Since item response theory centres on the measurement properties of individual items, rather than the instrument as a whole, it is not essential for all respondents to be examined using all items when using an item bank. It is even possible to select the 'best' items for individual patients using computerised adaptive testing algorithms [4]. This can reduce the burden of testing considerably for both patients and researchers. Furthermore, results from studies using different selections of items can be directly compared. Item banks measuring concepts such as quality of life [2,5], the impact of headaches [6], fatigue [7,8] and functional status [9-12] have been described.

Before an item bank can be implemented, it is essential to calibrate it. During the calibration process, the measurement properties of the individual items and the item bank as a whole are investigated. In contrast to the procedures used when developing fixed length instruments, it is not essential to present all items to all respondents in the calibration sample. It is often more efficient to offer targeted sets of items to particular groups within the sample. The items in common between any two sets of items are known as anchors. This kind of design is known as an incomplete, anchored calibration design and allows all items and patients to be calibrated on the same scale [13]. These designs have been used widely in preparing item banks for educational testing and has had some recognition in the development of medical instruments [14,15]. Developments in psychometric theory mean that it is now possible to perform the same types of analysis on data resulting from incomplete designs, as on complete designs [16-18]. The consequences of the use of this kind of design in the development of the ALDS item bank have been discussed previously [14,19]. If the primary aim of a study is to estimate the parameters of the two-parameter logistic item response theory model, as in this paper, little statistical information can be obtained from patients, whose functional status is much higher or lower than the difficulty of the items, with which they are presented [14,19]. The respondents described in this paper were chosen to maximise the statistical information on, and hence minimise the standard errors of the estimates of, the parameters of the item response theory model. For this reason, they may not be representative of the populations described.

The Academic Medical Center Linear Disability Score (ALDS) item bank was developed to quantify functional status in terms of the ability to perform activities of daily life. The ALDS item bank covers a large number of activities, which are suitable for assessing respondents with a very wide range of functional status and many types of chronic conditions. The items were obtained from a systematic review of generic and disease specific functional health instruments [1]. The methodology used to develop the ALDS item bank, including the use of incomplete calibration designs, has been described in depth [14]. Other papers have examined technical [20] and practical [21] aspects of methods to deal with missing item responses and the use of a 'not applicable' response category for the items. It has been shown that some of the ALDS items may have different measurement characteristics for males and females and for younger and older respondents [22]. The first results showed that the ALDS item bank had acceptable psychometric properties in a residential care population [9].

This study expands the results described in previous papers by examining the measurement properties of a selection of ALDS items, judged to be clinically relevant for a range of medical specialities, in a mixed population. The sample of respondents consisted of: residents of supported housing, residential care or nursing homes; patients attending an outpatients clinic for the treatment of chronic pain; hospitalized stroke patients; or attending an outpatients clinic for Parkinson's disease. These groups of patients were chosen because they have a broad range of chronic conditions and levels of functional impairment.

## Methods

### Respondents

A total of 1002 respondents were included. The respondents were previously described [9] residents of supported housing, residential care or nursing homes (551 respondents – 55%) and patients included in a number of studies in the Academic Medical Center, Amsterdam, the Netherlands. The studies were to examine: the effectiveness of treating patients with chronic pain in a specialised outpatients' clinic (235 – 23%); the effectiveness of treatment of stroke in an academic setting (127 – 13%); the progression of Parkinson's disease when only standard medication is prescribed (89 – 9%). The median age of the whole sample was 78 years (range 19 to 103 years) and 691 (69%) were female. Since the respondents described in this paper were chosen to minimise the standard errors of the estimates of the parameters in the item response theory model, they may not be truly representative of the populations described. This is particularly true for the residents of supported housing, residential care or nursing homes and for the stroke patients.

### Items

Each item in the ALDS item bank describes an activity of daily life. Examples include: 'Walking for more than 15 minutes'; 'showering'; and 'washing up'. The items were obtained from a systematic review of generic and disease specific instruments designed to measure functional health status [1]. Respondents were asked whether they could carry out each activity on their own at the present time. Each item has two response categories: 'I could carry out the activity' and 'I could not carry out the activity'. Two response categories were used because it has been shown that this maximises the reproducibility of scoring between time points and interviewers and increases clinical interpretability [23]. If a respondent had never had the opportunity to experience an activity, a 'not applicable' response was recorded. In the analysis, responses in the 'not applicable' category were treated as if the individual items has not been presented to the patients [21].

During the data collection, the interviewers signaled that some items were too 'hospital' based ('*washing oneself in bed*' for patients living at home or in residential care), had become 'old-fashioned' ('*using a public telephone*' and '*using a carpet beater*') or were so alike that respondents could not differentiate between them ('*showering and washing ones hair*' and '*showering, but not washing ones hair*'). For this reason, all of the 170 items included in the data collection were re-evaluated by two of the authors (NW and RJdH). A total of 115 items were judged to be actually suitable for inclusion in the ALDS item bank.

### Data collection

**Figure 1:**
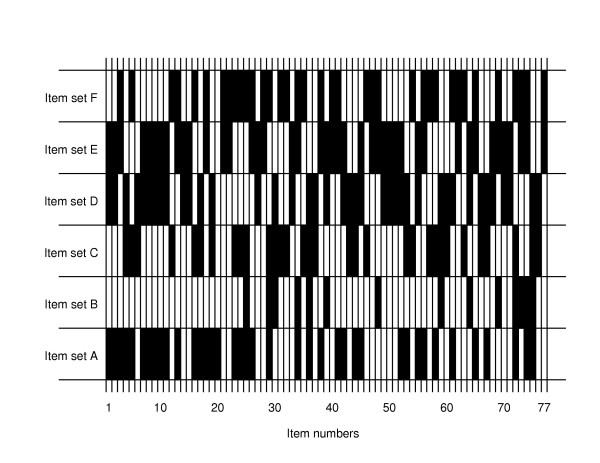
The items in each of the item sets *A *to *F*.

The respondents attending a clinic for chronic pain filled in a questionnaire with a single set of 88 items (set *A*). These items were chosen by one of the authors (MGWD) because they were clinically relevant for this patient population and spanned the whole range of functional status represented by the ALDS item bank. All other respondents were interviewed by specially trained nurses or doctors. The respondents who had had a stroke were all presented with a single set of 21 items chosen by one of the authors (NW) to cover the lower end of the ALDS item bank (set *B*). The residents of supported housing, residential care or nursing homes and the respondents with Parkinson's disease were presented with one of four sets of 80 items (sets *C*, *D*, *E *and *F*), which were described previously [9]. In these sets, each of 160 items covering the whole range of levels of functioning represented by the item bank was randomly allocated to two sets. Items sets *C *and *D *have half their items in common, as do sets *D *and *E*, sets *E *and *F *and sets *F *and *C*. The data collection design is illustrated in Figure 1. The items that are in each set are indicated by the solid blocks. It can be seen that all sets except *B *contain items from the whole range of the item bank and that set *B *mainly contains items, which are from the lower end of the range of functional status represented by the ALDS item bank. Further details of the item sets are given in Table 1. The the items that are in each set and the number of respondents, to whom each item was presented and the number responding in each category are indicated in Table 2.

**Table 1:** characteristics of the 6 sets of items

Item set	*n*	Population	Total number of items	Number of clinically relevant items	Number of items after analysis	Cronbach's alpha coefficient	Number of latent roots > 1	Variance explained by a single factor
*A*	235	Chronic pain	88	58	39	0.94	3	64%
*B*	127	Stroke	21	19	14	0.92	1	77%
*C*	179	RC + PD*	80	52	32	0.96	2	77%
*D*	164	RC + PD*	80	54	41	0.96	2	73%
*E*	157	RC + PD*	80	55	43	0.97	3	72%
*F*	140	RC + PD*	80	55	36	0.96	3	75%

All	1002		170	115	78	0.98	5	77%

*n *denotes the number of patients, who were presented with the item set

* RC denotes residents of supported housing, residential care or nursing homes

* PD denotes Parkinson's disease

**Table 2:** the 77 items and their measurement properties

				Number of responses						
Item	Item description	Item sets	Presented to	NA	can not	can	*β*	s.e.(*β*)	*α*	s.e.(*α*)
1	Cycling for 2 hours	ADE	556	22	448	86	-3.057	0.374	2.450	0.326
2	Vacuuming a flight of stairs	ADE	556	21	387	148	-2.653	0.307	3.231	0.399
3	Walking upstairs with a bag	AEF	532	13	364	155	-2.140	0.265	2.702	0.325
4	Cleaning a bathroom	ACD	578	10	368	200	-1.959	0.188	3.071	0.332
5	Vacuuming a room (furniture)	ACF	554	7	369	178	-1.879	0.166	2.455	0.223
6	Fetching groceries for 3–4 days	CD	343	0	267	76	-1.633	0.246	2.439	0.456
7	Going for a walk in the woods	ADE	556	17	345	204	-1.504	0.172	2.562	0.284
8	Traveling by bus or tram	ADE	556	18	307	231	-1.230	0.145	2.864	0.277
9	Walking for more than 15 min	ADE	556	2	298	256	-0.818	0.105	2.131	0.214
10	Carrying a tray	ADE	556	12	316	228	-0.808	0.100	1.618	0.163
11	Walking up a hill/high bridge	ADE	556	17	294	245	-0.781	0.094	1.993	0.165
12	Shopping for clothes	CF	319	2	206	111	-0.723	0.167	3.401	0.570
13	Cutting toe nails	AEF	532	4	286	242	-0.655	0.089	1.626	0.148
14	Filling a form in	DE	321	3	225	94	-0.614	0.088	1.028	0.131
15	Going to a party	DE	321	1	215	105	-0.560	0.092	1.407	0.171
16	Standing for 10 minutes	ACF	554	6	274	274	-0.525	0.090	1.834	0.161
17	Going to a restaurant	ACD	578	12	272	294	-0.481	0.085	1.975	0.173
18	Sweeping a floor	AEF	532	11	235	286	-0.450	0.105	2.872	0.336
19	Hanging up the washing	ACD	578	29	261	288	-0.445	0.092	2.257	0.248
20	Vacuuming a room	A	235	7	50	178	-0.347	0.203	2.470	0.546
21	Moving a bed or table en	EF	297	1	184	112	-0.304	0.091	1.342	0.144
22	Using a washing machine	DE	321	8	183	130	-0.234	0.106	2.072	0.271
23	Reaching into a high cupboard	ACF	554	5	248	301	-0.234	0.071	1.525	0.145
24	Walking up stairs	ACF	554	5	233	316	-0.192	0.082	2.190	0.241
25	Going to a bank or post office	ABCF	681	2	328	351	-0.130	0.089	3.119	0.305
26	Walking down stairs	AEF	532	4	204	324	-0.020	0.086	2.620	0.325
27	Going to a doctor	DE	321	9	164	148	0.020	0.125	3.289	0.435
28	Using a dustpan and brush	EF	297	2	159	136	0.083	0.108	2.503	0.422
29	Going for a short walk	ABCF	681	3	309	369	0.071	0.074	2.059	0.171
30	Writing a letter	BCD	470	4	245	221	0.175	0.068	0.862	0.092
31	Changing the sheets on a bed	CF	319	3	154	162	0.209	0.093	1.560	0.218
32	Crossing the road	CF	319	0	165	154	0.224	0.142	2.906	0.318
33	Opening a window	DE	321	0	149	172	0.240	0.086	1.417	0.179
34	Fetching groceries for 1–2 days	ABEF	659	2	276	381	0.291	0.088	2.529	0.230
35	Polishing shoes	CF	319	11	146	162	0.342	0.107	1.899	0.267
36	Showering	ABCD	705	6	243	456	0.657	0.077	1.950	0.183
37	Folding up the washing	CD	343	13	122	214	0.698	0.113	1.595	0.205
38	Dusting	AEF	532	18	141	373	0.702	0.100	2.391	0.267
39	Putting lace up shoes on	BDE	448	1	193	254	0.759	0.097	1.584	0.180
40	Cleaning a toilet	EF	297	1	115	181	0.779	0.122	2.102	0.293
41	Cutting finger nails	AEF	532	2	113	417	0.901	0.092	1.519	0.153
42	Making a bed	ADE	556	4	127	425	0.842	0.087	1.732	0.196
43	Reaching under a table	CD	343	1	104	238	0.918	0.103	1.438	0.171
44	Heating tinned food	ACD	578	10	143	425	0.922	0.107	2.572	0.265
45	Frying an egg	ADE	556	8	134	414	1.022	0.134	3.083	0.378
46	Reaching into a low cupboard	CF	319	0	90	229	1.092	0.134	1.513	0.206
47	Moving between two low chairs	EF	297	1	76	220	1.144	0.139	1.381	0.197
48	Picking something up	BEF	424	0	170	253	1.151	0.141	2.019	0.228
49	Cleaning a bathroom sink	DE	321	6	107	208	1.180	0.174	2.783	0.451
50	Putting the washing up away	DE	321	14	89	218	1.263	0.145	2.001	0.274
51	Reading a newspaper	DE	321	1	56	264	1.278	0.135	0.902	0.144
52	Getting in and out of a car	ADE	556	7	98	451	1.339	0.141	2.174	0.239
53	Making porridge	ACD	578	20	110	448	1.369	0.144	2.441	0.283
54	Clearing a table after a meal	CF	319	0	95	224	1.471	0.225	2.555	0.427
55	Peeling an apple	ADE	556	8	62	486	1.498	0.112	1.200	0.122
56	Making breakfast or lunch	AEF	532	8	87	437	1.517	0.173	2.273	0.300
57	Cleaning kitchen surfaces	CF	319	2	90	227	1.765	0.249	2.955	0.462
58	Putting a chair upto the table	ACF	554	3	75	476	1.777	0.186	2.060	0.277
59	Eating a meal at the table	BCD	470	0	101	369	1.788	0.149	1.352	0.134
60	Washing up	CD	343	1	74	268	1.863	0.223	2.244	0.309
61	Putting step-in shoes on	ADF	539	2	58	479	1.930	0.208	1.899	0.277
62	Sitting up in bed	EF	297	0	34	263	1.948	0.219	1.248	0.197
63	Getting a book off the shelf	CF	319	0	45	274	2.106	0.264	1.672	0.250
64	Answering the telephone	BDE	448	0	60	388	2.148	0.179	1.156	0.123
65	Hanging clothes up	AEF	532	5	66	461	2.192	0.248	2.645	0.369
66	Making coffee or tea	CD	343	0	58	285	2.348	0.298	2.316	0.332
67	Putting trousers on	ACD	578	5	70	503	2.376	0.261	2.744	0.364
68	Making a bowl of cereal	DE	321	2	55	264	2.280	0.297	2.292	0.335
69	Sitting on the edge of the bed	BEF	424	1	52	371	2.674	0.298	1.452	0.183
70	Moving between 2 dining chairs	DE	321	0	44	277	2.722	0.463	2.353	0.470
71	Washing lower body	DE	321	0	57	264	2.777	0.470	3.027	0.587
72	Putting a coat on	ABCF	681	3	99	579	2.859	0.308	2.392	0.291
73	Washing face and hands	BEF	424	0	75	349	2.969	0.389	2.067	0.284
74	Getting out of bed into a chair	ABEF	659	4	85	570	2.987	0.266	2.261	0.241
75	Going to the toilet	ABCD	705	5	115	585	3.077	0.461	2.954	0.453
76	Washing lower body (taken)	CD	343	1	52	290	3.235	0.580	3.140	0.616
77	Putting a T-shirt on	EF	297	0	32	265	3.494	0.960	2.690	0.792

### Statistical analysis

The statistical analysis is has been developed from previous work [14] and very similar to that in a previous paper [9]. The analysis concentrates on the two-parameter logistic item response theory model [24]. This model has been chosen because it allows a more realistic model [25] for the data to be built than when the more restrictive one-parameter logistic model [26]. In addition, the one-parameter logistic model has been shown to be unsuitable as a final model for describing data resulting from functional status items [9,14]. In the two-parameter logistic item response theory model, the probability, *P*_*ik*_, that patient *k *responds to item *i *in the category 'can' is modeled using



where *θ*_*k *_denotes the ability of patient *k *to perform activities of daily life. The discrimination parameter (*α*) and the difficulty parameter (*β*) describe the measurement characteristics of item *i*.

In step (a) items were excluded from further analysis if the item had been presented to fewer than 200 patients or if fewer than 10% or more than 90% of the responses were in the category 'can carry out'. In step (b), the items were examined using the one parameter logistic item response theory model [26] to investigate whether the item difficulty parameter (*β*_*i*_) was similar for male and female and for younger and older patients. This model was chosen because the parameters can be estimated using a smaller number of patients than are required to estimate the parameters in the two-parameter model satisfactorily [17]. The cutoff point between younger and older patients was 78 years, the median age. Items were excluded from further analysis if the difference in the value of the item difficulty parameters was more than half of the value of the standard deviance of the underlying distribution of ability parameters (*θ*). This is equivalent to a moderate effect size [27].

In step (c), estimates of the item parameters (*α*_*i *_and *β*_*i*_) were obtained. The fit of the model to the data from each item was assessed using *G*^2 ^statistics [17]. Items, for which the fit statistic had a *p*-value of less than 0.01 were excluded from further analysis. In step (d), the dimensionality of the item bank was examined using item response theory based full information factor analysis [9,16,17]. An exploratory factor analysis was carried out on each of the six item sets. To examine the population as a whole, a confirmatory factor analysis was carried out using the data from all 1002 respondents. In addition, Cronbach's coefficient alpha was calculated for each of the six item sets and for all of the data [18,28]. Steps (a), (b) and (c) were carried out in Bilog, version 3.0 [17] using marginal maximum likelihood estimation techniques with an empirically obtained distribution of the person parameters (*θ*). Step (d) was carried out using TESTFACT, version 4.0 [17].

## Results

**Figure 2:**
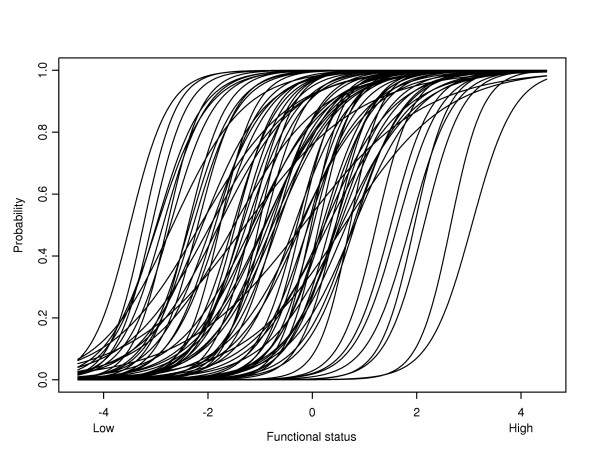
The probability that respondents with a range of levels of functional status can perform the items.

**Figure 3:**
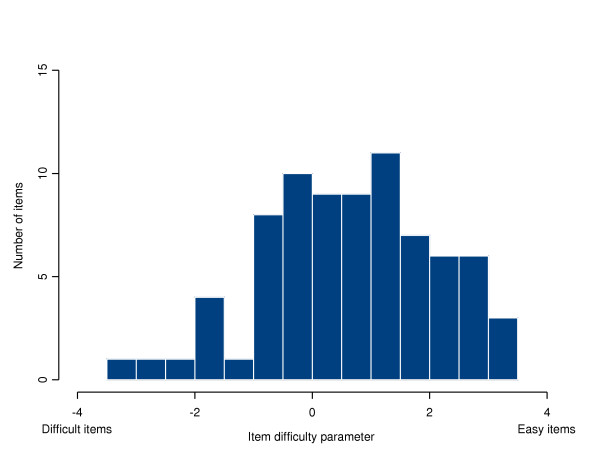
A histogram of the values of the difficulty parameters (*β*_*i*_).

Of the 115 items that were regarded as suitable for inclusion in the ALDS item bank, 38 were removed from and 77 were retained in the item bank. In step (a), a total of 24 items were removed from further analysis. Two items had been presented to fewer than 200 respondents, 1 item had fewer than 10% of responses in the category 'can carry out' and 21 items had more than 90% of responses in the category 'can carry out'. In step (b), a total of 11 items were removed from further analysis. Four items had different measurement characteristics for younger and older patients. Seven items had different measurement characteristics for male and female patients. In step (c), 3 items were removed from further analysis because their item fit statistic had a *p*-value less than 0.01. The item parameters (*α *and *β*) are given, with their standard errors, in Table 2. The probability that respondents with a range of levels of functional status can perform the items is illustrated in Figure 2. A histogram of the values of the difficulty parameters (*β*_*i*_) is given in Figure 3. It can be seen that the items cover the whole range of functioning, although there are more 'easy' than 'difficult' items.

In step (d), the values of Cronbach's coefficient alpha varied between 0.92 and 0.97 for the six sets of items and was equal to 0.98 for the whole data set. The values for each set of items are given in Table 1. The data sets had between 1 and 3 latent roots larger than 1 and the whole data set had 5 latent roots larger than 1. In general, there was one very large latent root and a number marginally greater than one. The percentage of variance explained by a single factor varied between 64% and 78% for the item sets and was equal to 77% for the whole data set.

## Discussion

In this study, an item response theory analysis of the ALDS item bank has been examined using an incomplete calibration design and a sample of 1002 respondents from: supported housing schemes, residential care or nursing homes (551); an outpatients' clinic for patients with chronic pain (235); following a stroke (127); and an outpatients clinic for Parkinson's disease (89). Each item in the analysis was presented to between 297 and 705 respondents. This is well above the minimum, of 200 respondents, regarded as necessary to implement the two-parameter logistic item response theory model [17].

The resulting item bank contains 77 items representing a wide range of levels of functional status. Although there are a number of items, which have very similar item parameters or content, there is no need to reduce the number of items further. Since estimates of respondents' functionals status are comparable, even when different sets of items are used to score them, researchers can choose items, which are particularly relevant to their 'population'. In this way, accurate estimates can be obtained, whilst minimising the burden of testing on both researchers and participants in clinical studies.

Before the item response theory based analysis was carried out, 55 of 170 items included in the data collection design were removed from the item bank because they were judged to be unsuitable for inclusion in the ALDS item bank. The insight required to judge that some of the items were unsuitable for the ALDS item bank could only been obtained once the items had been presented to a wide range of respondents. In the future, when developing an entirely new item bank, it may wise to conduct a broad pilot study before embarking on the full calibration study.

Previous results have shown that a proportion of items in the ALDS item bank have different measurement properties for men and women and for younger and older patients [9,22,29]. These results have been confirmed in this paper. Ideally, potential differences between the measurement characteristics of the items for different patient populations, for different groups of raters and for the interview and self-report versions of the ALDS item bank should also be examined in the same way as the differences between age and gender based groups. However, this was not possible for two reasons. Firstly, the groups of respondents with Parkinson's disease or acute stroke were too small to perform this analysis satisfactorily. Secondly, the levels of functioning in the respondents with chronic pain were much higher than those of the respondents living in residential care. This means that it was not possible to compare the groups at similar levels of functional status. Thirdly, all of patients in any given group were rated in the same way. Hence, it is not possible to separate differences caused by groups of raters and those caused by characteristics of the patient groups.

The respondents described in this paper were chosen to maximise the statistical information on, and hence minimise the standard errors of the estimates of, the parameters of the item response theory model. For this reason, they may not be representative groups from the populations described. This is particularly true for the residents of supported housing, residential care or nursing homes and for the stroke patients.

This does not have any consequences for the interpretation and implementation of the estimates of the parameters of the item response theory model [14] or the item response theory based factor analysis, but means that the values of Cronbach's alpha should be confirmed in future studies. In addition, the results for patients after a stroke and with Parkinson's disease need to be confirmed due to the small sample sizes used. Furthermore, in future studies it will be essential to examine whether the 77 items presented in this paper have the same measurement characteristics if they are presented to patients in an interview by nurses or by doctors or if patients respond to the items in a self-report situation.

The results presented in this article are different to those presented in a previous article examining the data from the residents of supported housing schemes, residential care or nursing homes [9]. There are two main reasons for this. Firstly, the selections of items included in the analysis were different. Secondly, the data described in this paper were collected from a mixed population of respondents. Previous research has commented on the differences between the one-parameter and two-parameter logistic item response theory models. In this paper, the two-parameter logistic item response theory model has been used. This model was chosen because previous results have shown that the one-parameter logistic model is unsuitable for this type of data [9].

## Conclusion

The results in this paper have shown that the ALDS item bank has promising measurement characteristics for a mixed patient population. The authors feel that the item bank can be used as a reliable indicator of functional health status in residents of supported housing, residential care or nursing homes, patients with chronic pain, acute stroke or Parkinson's disease. This paper has examined a mixed patient population, so the authors expect that the item bank will have good measurement characteristics for a wide range of other populations. However, care should be taken when using the ALDS item bank in other populations until these results have been confirmed.

Although this examination of the ALDS item bank has concentrated on six sets of items, future applications of the item bank are not bound to these sets of items. If these results are confirmed in future studies, the ALDS item bank will form a good foundation for a computerised adaptive testing procedure [4]. It would also be possible to select fixed length sets of items, specifically tailored to the level of functional status or clinical characteristics of a certain group of patients.

## Abbreviations

ALDS = Academic Medical Center linear disability score

## Competing interests

The author(s) declare that they have no competing interests.

## Authors' contributions

RL conceived the study and supervised the data collection in the residential care homes. NW supervised the data collection in the stroke population and MGWD in the chronic pain population. CAWG advised on the statistical analysis. RH carried out the statistical analysis and prepared the first draft and final version of the paper. NW, CAWG, RJdH, MGWD, MV and RL critically reviewed the manuscript.

## Funding

RH, NW and RL were supported by a grant from the Anton Meelmeijer fonds, a charity supporting innovative research in the Academic Medical Center, Amsterdam, The Netherlands.
